# Electrospun Poly(3-Hydroxybutyrate-Co-3-Hydroxyvalerate)/Olive Leaf Extract Fiber Mesh as Prospective Bio-Based Scaffold for Wound Healing

**DOI:** 10.3390/molecules27196208

**Published:** 2022-09-21

**Authors:** Jose Gustavo De la Ossa, Serena Danti, Jasmine Esposito Salsano, Bahareh Azimi, Veronika Tempesti, Niccoletta Barbani, Maria Digiacomo, Marco Macchia, Mohammed Jasim Uddin, Caterina Cristallini, Rossella Di Stefano, Andrea Lazzeri

**Affiliations:** 1 Cardiovascular Research Laboratory, Department of Surgical, Medical and Molecular Pathology and Critical Care Medicine, University of Pisa, 56126 Pisa, Italy; 2 Doctoral School in Life Sciences, University of Siena, 53100 Siena, Italy; 3 Department of Civil and Industrial Engineering, University of Pisa, 56122 Pisa, Italy; 4 Department of Pharmacy, University of Pisa, 56126 Pisa, Italy; 5 National Interuniversity Consortium of Materials Science and Technology (INSTM), 50121 Firenze, Italy; 6 Interdepartmental Research Center “Nutraceuticals & Food for Health”, University of Pisa, 56100 Pisa, Italy; 7 Photonics and Energy Research Laboratory, Department of Chemistry, University of Texas Rio Grande Valley, Edinburg, TX 78539, USA; 8 Institute for Chemical and Physical Processes (IPCF) @ Pisa, CNR, 56126 Pisa, Italy

**Keywords:** scaffold, bio-based, bioactive, fibroblasts, keratinocytes, ulcers, tissue engineering, biodegradation, polyphenols

## Abstract

Polyhydroxyalkanoates (PHAs) are a family of biopolyesters synthesized by various microorganisms. Due to their biocompatibility and biodegradation, PHAs have been proposed for biomedical applications, including tissue engineering scaffolds. Olive leaf extract (OLE) can be obtained from agri-food biowaste and is a source of polyphenols with remarkable antioxidant properties. This study aimed at incorporating OLE inside poly(hydroxybutyrate-co-hydroxyvalerate) (PHBHV) fibers via electrospinning to obtain bioactive bio-based blends that are useful in wound healing. PHBHV/OLE electrospun fibers with a size of 1.29 ± 0.34 µm were obtained. Fourier transform infrared chemical analysis showed a uniform surface distribution of hydrophilic -OH groups, confirming the presence of OLE in the electrospun fibers. The main OLE phenols were released from the fibers within 6 days. The biodegradation of the scaffolds in phosphate buffered saline was investigated, demonstrating an adequate stability in the presence of metalloproteinase 9 (MMP-9), an enzyme produced in chronic wounds. The scaffolds were preliminarily tested in vitro with HFFF2 fibroblasts and HaCaT keratinocytes, suggesting adequate cytocompatibility. PHBHV/OLE fiber meshes hold promising features for wound healing, including the treatment of ulcers, due to the long period of durability in an inflamed tissue environment and adequate cytocompatibility.

## 1. Introduction

Polyhydroxyalkanoates (PHAs) are biopolyesters synthesized by various microorganisms in the form of granular inclusions within the cells and accumulated as energy storage materials. More than 90 genera of both Gram-positive and Gram-negative bacteria have been identified as PHA producers under both aerobic and anaerobic conditions [1,2]. Recently, PHAs have been investigated as biomaterials due to their excellent biocompatibility and biodegradability. The proposed applications of PHAs encompass medical and pharmaceutical devices, nanotechnology, tissue engineering scaffolds, cardiovascular patches, and neural conduits, as well as wound dressings [3]. As a very wide family of biopolymers, PHAs possess a diverse chemical structure, enabling different molecular interactions that give rise to a tunable range of material properties, including mechanical behavior and degradation kinetics [3,4]. Among PHAs, poly(hydroxybutyrate-co-hydroxyvalerate) (PHBHV), is a copolymer investigated in the biomedical field. The presence of hydroxyvalerate (HV) imparts better processability to the rigid polyhydroxybutyrate (PHB). As such, PHBHV shows great potential for fabricating biomedical micro- and nanocarriers, being highly soluble in organic solvents, such as chloroform and dichloromethane, and insoluble in water [5]. In fact, PHBHV can be processed from the molten state, blended, and electrospun using copolymer solutions [6,7]. Even though many conventional and non-conventional technologies are available to produce scaffolds, electrospinning offers several advantages in the field of tissue engineering. Indeed, this technique allows the simple and cost-effective production of ultrafine fiber meshes, which, for their nature, mimic the fibrous extracellular matrix (ECM) of biological tissues, usually formed by collagen and elastic fibers, with a similar size [8].

Electrospinning exploits a simple key principle: a charged polymer solution jet is formed and collected on a collector when the applied electrostatic charge overcomes the surface tension of the polymer solution. As such, this technique can operate at room temperature using an electric field as a driving force for fiber formation. For this reason, electrospinning is particularly suitable to incorporate bioactive molecules and drugs into polymeric fibers, including PHBHV [9]. In a typical setting, electrospun fibrous scaffolds are assembled into nonwoven networks, which are deposited with an anisotropic (i.e., random) fiber orientation using a static planar collector [10]. Such randomly deposited fibrous meshes have potential applications, such as temporary substitutes for skin tissue engineering, since they replicate the microstructure of the dermal connective tissue [11]. Moreover, the high surface area of electrospun fibrous scaffolds allows oxygen permeability at the wound site, making these scaffolds suitable substrates for wound dressings [12].

Due to their tunable properties and biotech origin, PHAs and specifically PHBHV are very competitive candidates to replace other materials, e.g., those produced by the petrochemical industry, and have received increasing attention in the medical field for the development of high purity polymers from cheap extraction and recovery methods [12,13].

Skin damage represents a healthcare challenge for the medical sectors, affecting several million people worldwide. Some of them may be long-standing and unresponsive to conventional therapy due to complications from comorbidities, such as diabetes or infection [14]. In fact, wound healing is a complex and dynamic process connecting a cascade of biological reactions initiated in response to an injury. The process mainly involves the interaction of immune and non-immune cells (i.e., endothelial cells, fibroblasts, and keratinocytes) with soluble mediators (e.g., cytokines and growth factors), and ECM components [15,16]. The rate of healing in acute wounds differs from that in chronic wounds and is also dependent on the immunological status of the patient [17]. For example, the healing of ulcers is a very complex process; several products have been made available to facilitate its resolution [18]. Tissue-engineered skin replacements have been proposed as novel approaches to aid the healing of difficult skin wounds, such as ulcers. The presence of some biomolecules and biomaterials that improve the expression of defensins in the epidermal cells of tissue-engineered skin could play an important role in supporting an uncomplicated healing process [19,20]. However, several factors affecting the specific tissue microenvironment, sustained by the perseverance of inflammation, still make ulcer healing an unmet clinical need, which deserves additional research [21]. Among the many biomolecules used in ulcer treatment, we focused on olive leaf extract (OLE), since it is rich with polyphenols, namely powerful antioxidant molecules with a protective effect against stress damage in tissues [22,23]. It incorporates hydrophilic phenolic compounds, mainly secoiridoid oleuropein (about 17% *w*/*w*%), along with other compounds, such as apigenine-7-*O*-glucoside, luteolin-7-*O*-glucoside, quercetin, and caffeic acid, present in lower amounts (<0.1% *w*/*w*%) [24]. OLE is obtained from olive tree leaves as an agri-food biowaste during pruning, which is usually burnt in plantations; as such, its use as a bioactive ingredient and food supplement is in line with green economy goals [25,26]. It is believed that OLE acts as a free radical scavenger by a synergy of its many polyphenols. Therefore, it is an important means for the local delivery of OLE inside ulcers, which simultaneously induce connective tissue regeneration.

This study aimed to obtain bioactive bio-based PHBHV/OLE composites in the form of fiber meshes to be useful in wound healing. OLE was prepared and characterized for the content of polyphenols via high performance liquid chromatography (HPLC). PHBHV/OLE ultrafine fiber meshes were produced by electrospinning a PHBHV/OLE solution in appropriate solvents. The presence and distribution of OLE on the fiber surface was assessed via Fourier transform infrared spectroscopy (FT-IR). An in vitro degradation study was carried out to understand the stability of the scaffolds in biofluids. Finally, the fiber meshes were cultured with human fibroblasts and keratinocytes to confirm their cytocompatibility. Producing bioactive and fully bio-based scaffolds for ulcer repair is expected to contribute to functional and eco-sustainable biomedical products for skin damage.

## 2. Materials and Methods

### 2.1. OLE Preparation

Olive leaves were obtained from *Olivastra seggianese* plantation. The collection was performed at CNR-IVALSA (Follonica, GR, Italy). Leaf collection was carried out manually in March 2019. Leaves were placed in liquid nitrogen and crushed manually with a mortar and pestle. Water was added to the leaf powder and then the mixture was homogenized using a sonicator (IKA-Ultra Turrax Digital homogenizer at 100 rpm) for 1 min and a vortex mixer for 30 s. After centrifugation at 4000 rpm for 5 min, at 25 °C, the water phase was filtered using a syringe with a 0.22 µm pore-sized polystyrene filter and collected using a freeze-drier (Labconco, Kansas City, MO, USA), thus obtaining OLE.

### 2.2. OLE Characterization

The total phenol (TP) content in OLE was determined using the Folin–Ciocalteu method and gallic acid as the standard equivalent (µg GAE/mg) (Merk, Darmstadt, Germany), following a reported procedure [27]. About 10 mg of OLE was dissolved in 10 mL of CH_3_OH-H_2_O (80:20 *v*/*v*) and then an aliquot of this solution (1 mL) was mixed with 0.25 mL of Folin–Ciocalteu reagent (FCR) and 1.5 mL of Na_2_CO_3_ water solution (20% *w*/*v*). Afterwards, distilled water was added to reach the volume of 10 mL, and the resulting mixture was kept for 45 min at a controlled temperature of 25 °C. Spectrophotometric analysis was performed at λ = 725 nm [28,29].

HPLC was carried out to identify and quantify the major phenolic compounds of the OLE, using a slightly modified method that was developed in our previous study [24]. The retention times and UV absorbance spectra of phenolic compounds present in OLE were compared to those of the commercial standard and quantified at 278 nm, using *p*-hydroxyphenyl acetic acid as the internal standard, according to the previously reported method [26]. Sample concentrations were subsequently determined by linear regression, as a standard practice. For each calibration curve, the correlation coefficients were >0.99. HPLC analysis was performed using Thermo Finnigan-Spectra System SCM1000 equipped with a Spectra System P2000 (Pumps), Spectra System UV2000, set to 280 nm, and using a Phenomenex Gemini reverse-phase C18 column (250 × 4.6 mm, 5 μm particle size; Phenomenex, Castel Maggiore, Italy). The mobile phase was a mixture of H_2_O/acetic acid (97.5:2.5 *v*/*v*) (called “A”) and acetonitrile (called “B”), following a published practice [24]. The system was programmed as follows: a linear gradient was run from 5% B to 25% B for 20 min, followed by 50% B for 20 min, then 80% B for 10 min, and finally, re-equilibration to initial composition for 5 min. The flow rate used was 1 mL/min and the injected volume was 50.0 μL. Samples were injected as a mixture of methanol (MeOH; from Sigma-Aldrich)/phosphate buffer saline (PBS; from Sigma-Aldrich) (1:1 *v*/*v*). To assess the phenol content, 10 mg of OLE powder was dissolved in PBS and subsequently analyzed with HPLC.

Solvents used for HPLC analysis were purchased from Sigma Aldrich. Tyrosol, hydroxytyrosol, caffeic acid, and a *p*-coumaric acid, as analytical standards, were purchased from TCI (Zwijndrecht, Belgium). Oleuropein and *p*-hydroxyphenylacetic acid were purchased from Sigma-Aldrich. Luteolin-7-*O*-glucoside, apigenin-7-*O*-glucoside, and rutin were purchased from Extra synthese (Lyon, France). PBS for each analysis was diluted 10 times.

### 2.3. Fabrication of Electrospun PHBHV/OLE Fiber Meshes

PHBHV (with HV content 12% *w*/*v*%) was purchased from Sigma-Aldrich (Milan, Italy). The solution was prepared by mixing dichloromethane (DCM; from Carlo Erba (Rodano, MI, Italy) and MeOH (10:1 *w*/*w*). OLE at 16.5% *w*/*w*, with respect to PHBHV, was dissolved in the solvent mixture by magnetic stirring overnight at room temperature. The obtained PHBHV/OLE solution in DCM/MeOH was poured into a 10 mL glass syringe and placed into a syringe pump (NE-300, New Era Pump Systems, Inc., Farmingdale, NY, USA). The ground terminal of high voltage supply (S1600079 Linari High Voltage, Linari Engineering s.r.l., Pisa, Italy) was connected to the metal needle, whereas the positive terminal was connected to the collector. The fibers were collected on a rotatory collector set at quasi-zero velocity on aluminum foil to collect randomly oriented fibers with a uniform mesh thickness. The fiber mesh was obtained using a tip-to-collector distance of 15 cm at a flow rate of 0.0016 mL/min and 30 kV. The mesh was electrospun for 1 h to obtain self-standing samples. Humidity was 40% and temperature was 20 °C throughout. PHBHV fiber meshes without OLE were collected in the same way to be used as controls. Image J software (version 1.52t) was used to evaluate the size of fibers. An average of 100 measurements was taken for each sample. After production, the fibers were left dry under a laminar hood and stored at 4 °C for further use to preserve phenolic compound activity. Additionally, Image J was used to evaluate the percentage of the porosity of the fibers.

### 2.4. Characterization of PHBHV/OLE Electrospun Fibers

The morphology of the fiber meshes was evaluated using scanning electron microscopy (SEM) using a FEI FEG-Quanta 450 instrument (Field Electron and Ion Company, Hillsboro, OR, USA). The samples were sputtered with gold (Gold Edwards SP150B, UK) before analysis. FT-IR in attenuated total reflectance (ATR) mode was used to characterize the presence of specific chemical groups in the tested samples. Analysis of the bulk samples was performed using Nicolet T380 instrument (Thermo Scientific, Waltham, MA, USA) equipped with a Smart ITX ATR attachment with a diamond plate.

FT-IR analysis Chemical Imaging was then used to evaluate the surface distribution of components in the biomaterial, obtaining chemical and correlation maps to visualize their distribution. The micro-ATR (µATR) and spotlight FT-IR spectra maps were carried out by means of a Perkin Elmer Spectrum One FTIR Spectrometer, equipped with a Universal ATR Sampling Accessory and a Spectrum Spotlight 300 FT-IR Imaging System (Perkin Elmer, Norwalk, CT, USA), using the image mode of the instrument. For each sample, areas of 1 mm × 1 mm were analyzed. Afterwards, an IR image was generated using a nitrogen-cooled, 16-pixel mercury cadmium telluride line detector at a resolution of 25 µm per pixel. An absorbance spectrum was recorded for each pixel in the µATR mode. Spotlight software used for the acquisition was also employed to pre-process the spectra. Consequently, the spectra images were analyzed with a reference correlation image. Additionally, the full spectral maps were analyzed by principal component analysis (PCA). For this analysis, the data set was composed of the spectra that make up the full spectral image. The spectral data grouping of similar variability is represented by the same color, allowing the identification of different spectral groups.

The quantity of phenolic compounds incorporated inside the polymer fibers was investigated using 4 cm^2^ square meshes incubated in MeOH and the resulting solution was analyzed by HPLC, as described in Section 2.2.

### 2.5. Degradation Study of PHBHV/OLE Electrospun Fibers

A complete degradation analysis was carried out in vitro on PHBHV/OLE electrospun fibers. The tests were conducted by soaking samples of the fiber meshes (size 1 × 1 cm^2^; weight 5 mg) in PBS and placing them in a 37 °C oven for up to 2 months. To understand the sample susceptibility to enzymatic degradation, a set of samples (*n* = 4) were soaked in PBS with metalloproteinase 9 (MMP-9; from Sigma-Aldrich), at 10 ng in 200 µL of PBS for a final volume of 1.5 mL per sample, at room temperature. The solution was replaced every week. Every week, the samples were carefully removed from the solutions, dried under a laminar flow hood for 2 h, and weighted in an analytic balance (AS 220.R2, RADWAG, Poland). The morphology of the samples was observed via SEM. The analysis of degraded samples was also investigated by molecular loss mass, using GPC (Perkin Elmer, USA). Chloroform was used as the mobile phase and its constant flow-rate was maintained at 1.0 mL/min. Polymer sample solutions were prepared by dissolving samples (0.5% *w*%) in chloroform (1 mL) and sterile filtering (0.45 µm filters). A total volume of 100 µL was used for GPC analysis. Calibration was performed using the standards of polystyrene to determine the number-average and weight-average molecular weights (Mn and Mw) of samples.

### 2.6. In Vitro Phenol Release Study

To investigate the release of phenols from PHBHV/OLE, the diffusion of phenolic compounds in a release medium (i.e., PBS) was determined. A 4 cm^2^ square of PHBHV/OLE was inserted into a 3 cm diameter well of a multi-well plate and 2 mL of PBS was added to each well. Then, the plate containing the solutions was incubated in an oven at a controlled temperature of 37 °C. At defined time intervals (0, 0.5, 1, 1.5, 2, 2.5, 3, 4, 6, 24, 48, 72, 144 h), the supernatant was removed, the well was washed with 1 mL of PBS and an equal amount of the fresh PBS (2 mL) was replaced each time [30,31]. The qualitative-quantitative evaluation of the phenols released from the polymer was carried out by HPLC analysis, following a previously reported method [32].

### 2.7. Cytocompatibility of PHBHV/OLE Fiber Scaffolds

Human Caucasian foreskin fetal fibroblasts (HFFF2) (ICLC #Catalog HL95002), were kindly provided from IRCCS AOU San Martino, National Institute for Cancer Research, Italy. The cells were cultured with Dulbecco’s Modified Eagle Medium (DMEM) with 10% fetal bovine serum (FBS), 2 mM L-Glutamine, and 1% penicillin-streptomycin solution (all from Lonza, Walkersville, MD, USA) in T75 tissue culture flasks (Euroclone S.p.A, Pero, MI, Italy). The cells were seeded at 2 × 10^4^ cells/cm^2^ in 75 cm^2^ in tissue culture polystyrene (TCPS) flasks to allow expansion. Cell cultures were performed in a humidified incubator set at 37 °C in 5%CO_2_/95% air.

The scaffolds were cut to measure 1 × 1 cm^2^ and sterilized under UV light for 30 min. Before seeding, the scaffolds were soaked with a sterile-filtered 2% (*w*/*v*) gelatin (type B, from bovine skin; Sigma Aldrich) aqueous solution by soaking for 30 min to facilitate cell adhesion. Excess gelatin solution was removed before cell seeding. HFFF2 cells were detached from the TCPS flasks using trypsin 0.25% (*v*/*v*%)/ethylenediaminetetraacetic acid (EDTA) (Euroclone S.p.A.) and seeded on PHBHV and PHBHV/OLE fiber meshes at a density of 2 × 10^5^ cells/20 µL in culture medium per scaffold, placed in a 24-well plate. Cell/scaffold constructs were cultured in 1 mL of culture medium replaced every 2 days for 1 week. At the endpoint, 72 h after seeding, the metabolic activity of cell/scaffold constructs was evaluated using resazurin dye (Sigma-Aldrich), dissolved at a concentration of 0.5 mg/mL in a culture medium and incubated with the samples for 4 h. The dye contains a REDOX indicator, turning from blue into pink, after being metabolized by cells. The absorbance (λ) of supernatants was measured with a spectrophotometer (Victor 3; PerkinElmer, Waltham, MA, USA) under a double wavelength reading (570 nm and 600 nm). Finally, the dye reduction percent (%red) was calculated, correlating the absorbance values and the molar extinction coefficients of the dye at the selected wavelengths. The equation applied is shown below (Equation (1)), in which *λ* = absorbance, *s* = sample, and *c* = control.

(1) $$\%{\mathrm{Dye}}_{\mathrm{red}}=100\cdot \frac{\left(117,216\cdot \lambda_{s\left(570\;nm\right)}-80,586\cdot \lambda_{s\left(600\;nm\right)}\right)}{\left(155,677\cdot \lambda_{c\left(600\;nm\right)}-14,652\cdot \lambda_{c\left(570\;nm\right)}\right)}$$

At the endpoint, the cell/scaffold constructs were washed two times with PBS and fixed in 1% (*w*/*v*) neutral buffered formalin for 10 min at 4 °C. Formalin-fixed samples were washed again with PBS and permeabilized with 0.1% (*v*/*v*) triton (Sigma) in PBS for 5 min, prior to being incubated with phalloidin Alexa Fluor 633 (Thermo Fisher Scientific) for 45 min at room temperature in the dark, following the manufacturer’s procedure. Counterstaining was performed by incubating the sample in 10 µg/mL 4′,6-diamidino-2-phenylindole (DAPI, Thermo Fisher Scientific, Waltham, MA, USA) in PBS for 10 min at room temperature in the dark. Specimens were observed using an inverted fluorescence microscope equipped with a digital camera (Nikon Eclipse Ti, Amsterdam, The Netherlands).

Cytocompatibility was also investigated using human keratinocytes. HaCaT cell line was obtained from CLS–Cell Lines Service, Eppelheim, Germany. Cells were expanded, as reported above for HFFF2, and finally seeded at 1 × 10^5^ cells/20 µL in culture medium per sterile scaffold and placed in 24-well plates as previously reported. Cell/scaffold constructs were cultured for 72 h. At the endpoint, the samples were fixed in 10% formalin for 10 min, rinsed in distilled water, and dried in a vacuum oven set at 37 °C. The specimens were mounted on aluminum stumps, sputter-coated via an argon gas carrier for 15 s (Coater SC7620, Quorum Technologies Ltd., West Sussex, UK), and observed by SEM.

### 2.8. Statistical Analysis

Statistical analysis was carried out by Microsoft Office Excel and shown as mean ± standard deviation (SD) using Student’s *t*-test. Probability (*p*) values < 0.05 were considered to be statistically significant differences.

## 3. Results

### 3.1. OLE Characterization

The TP content of OLE was estimated using the Folin–Ciocalteu method, resulting in 58.47 µg GAE/mg. Furthermore, HPLC analysis allowed the main polyphenols present in OLE to be determined, which were reported in Table 1. They were oleuropein and luteolin-7-*O*-glucoside, with a concentration of 32.64 ± 3.06 mg/g of OLE (3.26 *w*/*w*%) and 6.97 ± 0.24 mg/g of OLE (0.70 *w*/*w*%), respectively; moreover, OLE contained apigenin-7-*O*-glucoside with a concentration of 1.97 ± 0.17 mg/g of OLE (0.20 *w*/*w*%), rutin with a concentration of 3.37 ± 0.33 mg/g of OLE (0.34 *w*/*w*%), and other compounds (hydroxytyrosol, caffeic acid, and *p*-coumaric acid) with a concentration lower than 1 mg/g of OLE.

### 3.2. Characterization of PHBHV/OLE Fiber Meshes

**Figure 1:**
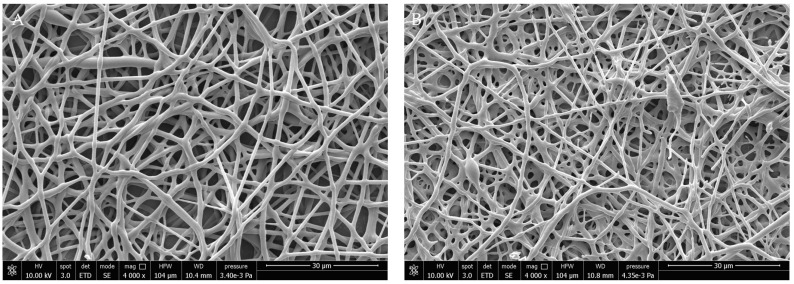
SEM micrographs of (**A**) PHBHV and (**B**) PHBHV/OLE electrospun fibers. Scale bar is 20 µm, 4000× magnification, 10 kV voltage.

Morphological properties of the PHBHV and PHBHV/OLE fibers were investigated via SEM. The fibrous meshes resulted in smooth fiber surfaces in both electrospun types. Additionally, the PHBHV/OLE fiber meshes showed differences in porosity with 42.22% in PHBHV and 63.33% in PHBHV/OLE. As analyzed via image J, the samples resulted in a mean fiber diameter of 1.29 ± 0.34 µm for PHBHV/OLE, while that for PHBHV was 0.93 ± 0.23 µm. Representative micrographs of PHBHV and PHBHV/OLE fibers are reported in Figure 1.

**Figure 2:**
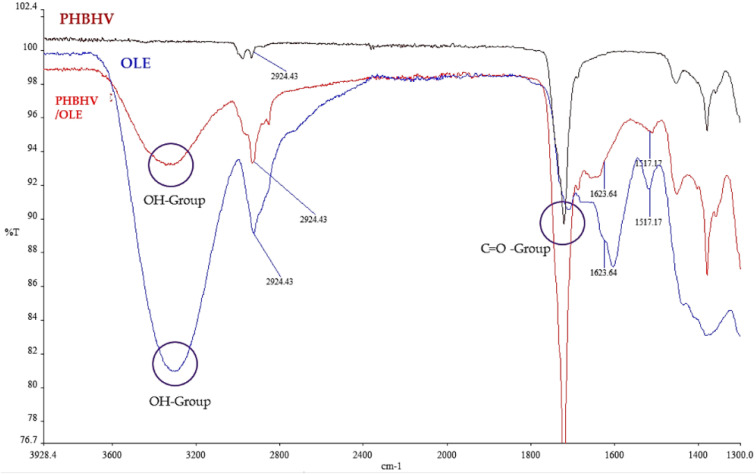
FT-IR ATR spectra of OLE, PHBHV, and PHBHV/OLE fibers showing characteristic bands. *Y*-axis is transmittance %, *x*-axis is wavenumbers (cm^−1^).

FT-IR ATR spectra, acquired for plain OLE, PHBHV, and PHBHV/OLE fiber samples are shown in Figure 2.

**Figure 3:**
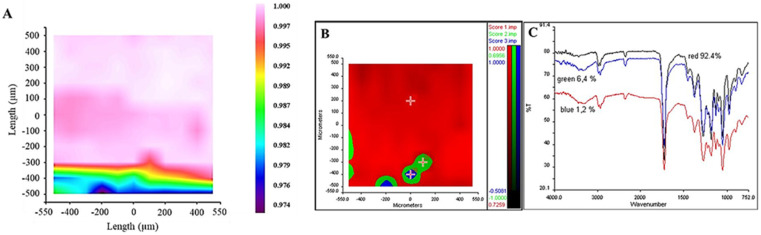
Results of chemical imaging analysis showing: (**A**) correlation map with the characteristic spectrum in the absorption region of the -OH groups. The correlation map shows values between 0.974 and 1.000 over the entire analyzed surface; (**B**) Statistical map elaborated by PCA to identify the zones on the map with the same spectral variability. The PCA analysis showed the presence of 3 spectral groups, identified in blue, green, and red; (**C**) Spectrum of each zone is represented in the graph of FT-IR spectra.

As evidenced in the spectra of OLE-containing samples, the band at 3300 cm^−1^ is indicative of molecular -OH groups related to the hydrophilicity of the extract due to the presence of phenols. In the spectra, the absorption bands between 1700–1738 cm^−1^, related to the C=O groups of PHBHV and OLE, were also indicated, which is confirmed in the literature [33]. The characteristic peak at 2925 cm^−1^ may be attributed to the C-H stretching node, which in OLE is present in the terminal methyl groups, while the peak of 1624 cm^−1^ is attributed to the presence of amide I. Chemical imaging maps acquired in FT-IR show the homogeneous presence of -OH groups, ascribable to OLE, on the mesh surface, thus indicating an optimally dispersed composite material (Figure 3).

A degradation analysis was carried out in vitro on PHBHV/OLE scaffolds, using PBS and PBS with MMP-9. The mass loss evaluated using an analytical balance up to 56 days is reported in Table 2. After 56 days, the average gravimetric weight loss percentage of fibers containing OLE after incubation in PBS was 4.21%, while after incubation in PBS with MMP-9, the weight loss reached 9.6% (Table 2).

**Figure 4:**
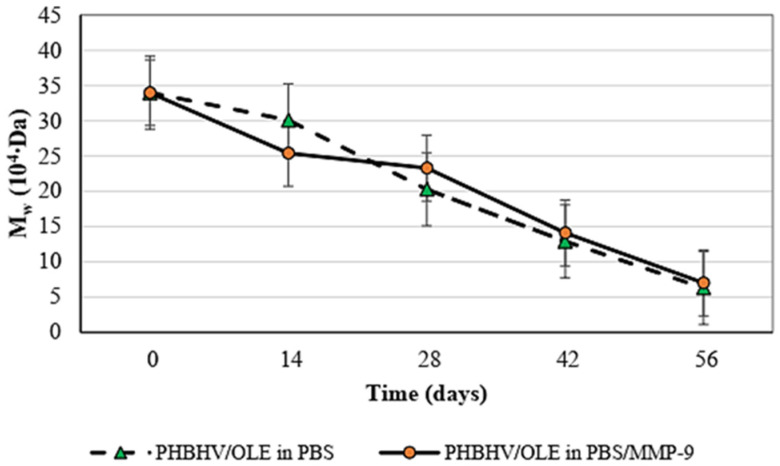
Graph showing molecular weight (M_w_) loss obtained from GPC of PHBHV/OLE fiber meshes for 8 weeks in different media: plain PBS and PBS added with MMP-9 at 50 ng/mL (*n* = 4).

**Figure 5:**
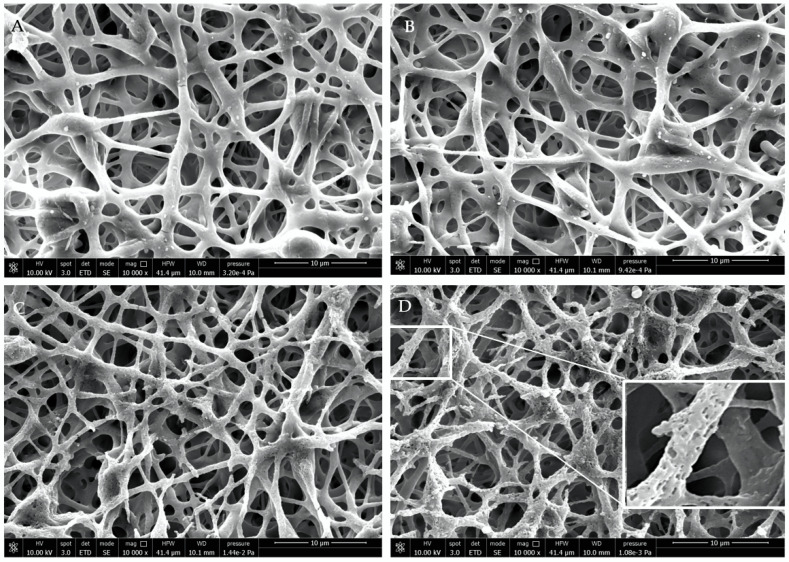
SEM micrographs of PHBHV/OLE under degradation in PBS for (**A**) 2 weeks, (**B**) 4 weeks, (**C**) 6 weeks, and (**D**) 8 weeks. Lens in (**D**) shows evidence of pore formation on the fiber surfaces. Scale bar is 10 µm, 10,000× magnification, 10 kV voltage.

The change of molecular weight of PHBHV/OLE fiber meshes after incubation in PBS with MMP-9 was evaluated by GPC. Molecular weight reduction as a function of time due to the breakdown of the copolymer chains was observed. In the first two weeks, the rupture of PHBHV chains was, on average but not significantly, higher when the fibers with OLE were incubated in PBS with the MMP-9 enzyme, whereas in the following weeks, the rate of degradation was similar in two incubation media, decreasing by about 28 × 10^4^ kDa (Figure 4). SEM morphological analysis is reported in Figure 5.

Morphological analysis showed gradual changes in the surface of the fibers including the formation of pores and cracks, particularly evident after 4 weeks (Figure 5C,D), while maintaining the mesh structure. This result is in agreement with the gravimetric outcomes, showing a partial bulk erosion lower than 10% after 8-week incubation in the presence of MMP-9.

The phenolic content of PHBHV/OLE obtained via HPLC analysis indicated that the amount of main phenols present was 21.84 ± 0.14 µg for oleuropein, 7.22 ± 0.78 µg for luteolin-7-*O*-glucoside, and 3.54 ± 0.13 µg for apigenin-7-*O*-glucoside.

### 3.3. OLE Release from PHBHV/OLE Fiber Meshes

**Figure 6:**
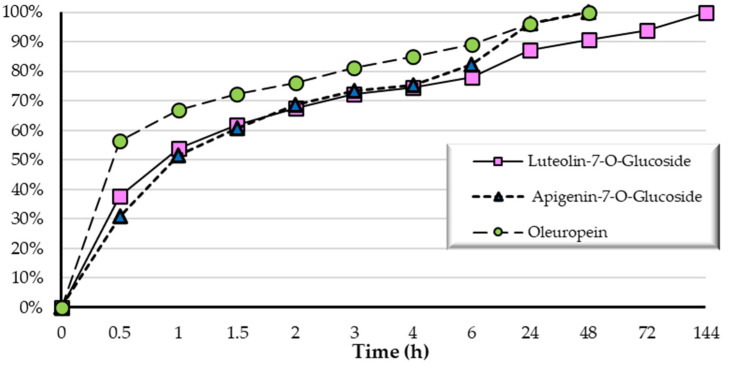
Graph showing the cumulative release (Release %) of polyphenols from PHBHV/OLE fiber meshes up to 144 h.

The release profile of oleuropein, luteolin-7-*O*-glucoside, and apigenin-7-*O*-glucoside from the copolymer was analyzed. The quantity of these released phenolic compounds during the submersion time, reported in Figure 6 and Table 3, are expressed as a percentage of the initial amount (reported above). After the first 30 min, the amount of oleuropein released was about 60% of the total oleuropein content in polymer. Then, the release became more gradual, reaching about 90% after 6 h. As it concerns luteolin-7-*O*-glucoside and apigenin-7-*O*-glucoside, after the first 30 min, the release was about 40% and 30%, respectively, and was about 80% after 6 h in both cases. Thus, the phenolic compounds incorporated with PHBHV/OLE were effectively released into the PBS medium.

### 3.4. Cytocompatibility of the Scaffolds Using HFFF2 Cells

**Figure 7:**
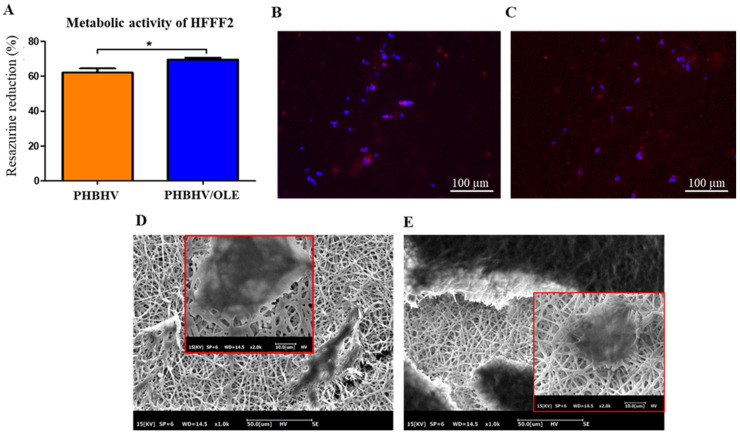
Cytocompatibility of PHBHV and PHBHV/OLE scaffolds. (**A**–**C**) The scaffolds were cultured with fibroblasts (HFFF2) for 3 days: (**A**) bar graph showing the metabolic activity of the cells after 72 h (*n* = 3; *t*-test; one way, * *p* < 0.01); (**B**,**C**) florescence staining of *f*-actin in red and nuclei in blue for (**B**) PHBHV and (**C**) PHBHV/OLE. The scale bar in (**B**,**C**) is 100 µm. (**D**,**E**) SEM micrographs of the scaffolds cultured with keratinocytes (HaCaT) for 3 days: (**D**) PHBHV and (**E**) PHBHV/OLE; scale bar is 50 µm. Lens showing details of cell attachment to the fibers; scale bar is 10 µm.

A cytocompatibility study of PHBHV and PHBHV/OLE was performed in vitro using HFFF2 and HaCaT cell lines. The results are shown in Figure 7. The resazurin test was performed at 72 h after seeding, when the release of oleuropein and apigenin-7-*O*-glucoside was complete and the release of luteolin-7-*O*-glucoside was at 94.01% (Figure 7A). By comparing to the pure copolymer fibers, PHBHV/OLE fiber scaffolds showed the highest metabolic activity of the HFFF2 fibroblasts, namely 69.5% ± 3.2% (*p* < 0.01). As the test was performed against negative controls (scaffolds + dye, without cells), the quantitative results are intended only for intra-sample comparison. The presence of cells adhered to the fibers is confirmed by fluorescence micrographs (Figure 7B,C).

Ultimately, cytocompatibility was tested by seeding HaCaT on the top of the fibrous meshes. SEM analysis showed cellular aggregates on both samples (Figure 7E), forming a thick cell layer in the sample containing the extract (Figure 7E).

## 4. Discussion

Chronic wounds, such as ulcers, represent an unsolved problem worldwide [14]. Concomitant factors, such as diabetes, sustain unhealed conditions for weeks and months by interfering with the natural repairing process, thus leading to open wounds prone to infections [15,16]. The search for new dressings able to support wound healing is still ongoing. It is expected that naturally derived materials and molecules can open new routes for managing chronic wounds [34]. For this purpose, we investigated a novel class of biopolymers, PHAs, blended with olive-leaf-derived polyphenols. Specifically, we prepared ultrafine fiber meshes made of PHBHV and OLE blends, having the morphological, physico-chemical, cytocompatibility, and functional properties that make them suitable scaffolds for skin repair. In fact, electrospinning of polymer blends is exploited to tune the scaffold properties to reach the desired features [35]. The extraction phase of OLE allowed a mixture of active principles to be obtained, which can be entrapped in the fibers to improve biological functions by releasing the biomolecules over time [14,36]. The procedure of electrospinning, including the set-up of the solvent system, was optimized to produce meshes of fibers, forming highly porous structures. In vitro tests carried out on PHBHV/OLE meshes demonstrated a long-term biodegradation, and an improved cytocompatibility with respect to OLE unloaded scaffolds. In addition, the electrospun mesh could serve as a biomolecule carrier for a controlled release of antioxidant principles able to treat human tissue alterations.

Concerning the physicochemical characterization of PHBHV and PHBHV/OLE by FT-IR, the analysis indicated an absorption band at 1700–1738 cm^−1^ that corresponds to the C=O group of PHAs in both samples and a band in 3300–3200 cm^−1^ that contains several vibration modes, present only in the sample with OLE, which is associated with hydroxyl groups including those of adsorbed water [33]. The latter band can be considered as an index of the material hydrophilicity degree, due to OLE incorporation into the PHBHV fibers. In addition, the chemical map associated with -OH groups confirmed the efficiency of the loading procedure of OLE and a homogeneous distribution of OLE over the whole mesh. The results strongly suggest that OLE can be present both on the surface and inside the PHBHV fibers. In fact, the FT-IR instrument used in ATR mode can detect the material composition up to a 1 µm depth, which is of the same order of magnitude of the diameter of the fibers (1.29 ± 0.34 µm).

An in vitro biodegradation study was carried out on the PHBHV/OLE samples for a period of two months by immersing the samples in PBS and PBS with MMP-9, aimed to resemble the conditions of a normal tissue remodeling process. It is known, in fact, that MMP-9 is a proteolytic enzyme highly expressed during the wound healing process and its proteolytic activity can allow ECM degradation, thus delaying wound healing [37]. At different time-points of incubation in two distinct media, the samples were analyzed by measuring the weight loss. The results show a slow degradation with a modest mass reduction at the endpoint, both in PBS (4.21%) and in PBS in the presence of MMP-9 (9.64%). In general, PHA films (i.e., PHB, PHBHV) are reported to have a slow hydrolytic degradation in vitro, namely only 5% of their initial weight after 240 days in PBS [37]. In our study, a slightly higher erosion was observed, which can be attributed to the hydrophilic contribution of OLE within the PHBHV fiber structure. In addition, the presence of OLE could favor the surface adsorption of MMP-9 molecules in the ester groups of PHBHV, which finally resulted in an increased degradation due to the combined enzymatic and hydrolytic effects. In this respect, GPC outcomes showed an evident reduction of molecular mass, thus indicating the breakdown of ester bonds in the polymeric chains without an evident bulk erosion, as also observed by SEM analysis. The trend of molecular weight change showed how the hydrolytic process was responsible for the rupture of copolymeric chains with a similar behavior in the two different media. The effect of the enzyme on the corresponding erosion process was therefore observed for longer durations as pointed out by the weight loss and morphological aspect of the PHBHV/OLE fibers after 8 weeks. Salguero et al. assessed the PHB degradation in vitro, using the same protocol performed in abiotic conditions, namely PBS at pH 7 and at 37 °C for 40 days [38]. After this period, their results showed a limited PHB mass loss, ranging in 1.0–1.5%. Finally, the use of an enzyme permitted an increased time control upon polymer degradation [38]. During the degradation, the enzymes break the long polymeric chain, forming oligomers and, after some time, these oligomers may be hydrolyzed to monomeric units of the polymer [39,40].

OLE showed a release profile in our PHBHV fiber samples that was differently tuned according to the diverse bioactive components. Indeed, biomolecules, such as oleuropein and apigenin-7-*O*-glucoside, were fully released from the PHBHV fibers at 48 h, while luteolin-7-*O*-glucoside were released at 144 h. Such a long-time release of polyphenols (4–6 days) shows potential for the application of the PHBHV/OLE fibrous meshes for wound healing, due to the properties of OLE. Patients suffering from skin ulcers—usually, secondarily to other pathologies, such as diabetes—have a delayed healing time; they may take drugs, either orally or directly applied to the skin [12]. The rapid release of the biomolecules in OLE and the topical action, enhanced by the large surface area provided by the electrospun fibrous material, can be considered an ideal strategy to deliver polyphenols as a “natural drug” to the damage site, especially in chronic wounds of the skin [7,41,42,43]. In our preliminary tests of cytocompatibility, human fibroblasts and human keratinocytes were able to grow on the PHBHV/OLE electrospun meshes in vitro. The former feature is particularly impellent in partial and full thickness wounds, thus fibroblast infiltration in electrospun scaffolds is highly desirable [44]. In addition, keratinocytes covered the scaffold surface through cellular layers, which is indicative of epithelization capacity. We have recently disclosed the immunomodulatory activity of other PHA/OLE fibers in vitro using human dermal keratinocytes occurring via the downregulation of the inflammatory pattern, thus proving the potential for skin re-epithelization and regeneration, which are secondary to inflammation resolution [45].

Lastly, even though the antimicrobial properties of OLE are still controversial, we have recently demonstrated that OLE can induce the expression of the antimicrobial peptide human defensin 2 (HBD-2) gene in human dermal keratinocytes, thus highlighting a potentially powerful effect in chronic wound repair [32]. Moreover, we have also unveiled new strategies to enhance the antimicrobial effect by combining OLE with other green technologies, such as cold atmospheric plasma [46].

The produced biofunctional ultrafine fibers may represent a bio-based scaffold which is sufficiently stable in an inflamed tissue environment, as demonstrated by its long degradation time, even in presence of MMP-9, showing promise for the medical application of a new generation of green wound dressings. Producing new natural-based biomaterials that are able to reduce inflammation, increase innate immune response, and provide a suitable architecture for dermal and epidermal tissue regeneration over a long time could greatly help the management of chronic wounds.

## 5. Conclusions

We demonstrated that OLE could be efficiently incorporated inside PHBHV electrospun scaffolds with a fiber size of 1.29 ± 0.34 µm, thus tuning the hydrophilic properties at the fiber surface. The PHBHV/OLE meshes highlighted a very long degradation time in vitro, showing a weight loss of 4.21% ± 0.14% in PBS and 9.64% ± 0.28% in PBS added with MMP-9 after 8 weeks. The copolymer molecular weight reduction was about 28 × 10^4^ kDa in both degradation media, and surface erosion phenomena due to nanopore formation on the fibers in PBS were detected. The release of the main polyphenols of OLE from the fibers was assessed, showing a release occurring within 4–6 days. Finally, the first observation with fibroblasts confirmed that the produced fibers were cytocompatible. Due to all of these characteristics, they appear to be promising in medical applications including wound healing and possibly skin ulcers. The utilization of PHBHV and OLE thus represents a prospective strategy in biomedicine, which is also compliant with sustainable use, a long period of durability, adequate biocompatibility, and environmental friendliness.

## Figures and Tables

**Table 1:** Content (mg/g) of main phenols in OLE (mg/g of OLE). Data are expressed as mean ± standard deviation (SD) (*n* = 2).

Hydroxy-Tyrosol	Caffeic Acid	*p*-Coumaric Acid	Rutin	Luteolin-7-*O*-Glucoside	Apigenin-7-*O*-Glucoside	Oleuropein
0.85 ± 0.08	0.18 ± 0.02	0.085 ± 0.007	3.37 ± 0.33	6.97 ± 0.24	1.97 ± 0.17	32.64 ± 3.06

**Table 2:** Gravimetric weight loss (mg/mg %) of PHBHV/OLE fibers in different media: PBS and PBS added with MMP-9. Data are expressed as mean ± SD (*n* = 4).

Time (days)	Weight Loss (mg/mg %) **in Degradation Media:**	
	**PBS**	**PBS + MMP-9**
12	2.76 ± 0.07	2.44 ± 0.07
28	3.33 ± 0.11	3.75 ± 0.11
42	3.85 ± 0.11	7.14 ± 0.02
56	4.21 ± 0.14	9.64 ± 0.28

**Table 3:** Cumulative release (µg) of the main OLE phenolic compounds in a 4 cm^2^ square of PHBHV/OLE fiber mesh. Data are expressed as means ± SD (*n* = 2).

Time (h)	Luteolin-7-*O*-Glucoside	Apigenin-7-*O*-Glucoside	Oleuropein
0	0.00	0.00	0.00
0.5	3.44 ± 0.85	0.95 ± 0.04	12.29 ± 1.30
1.0	3.48 ± 1,45	1.58 ± 0.49	14.53 ± 2.60
1.5	4.01 ± 1.58	1.86 ± 0.71	15.70 ± 2.96
2.0	4.37 ± 1.75	2.10 ± 0.94	16.53 ± 2.79
3.0	4.68 ± 1.95	2.25 ± 0.98	17.63 ± 2.52
4.0	4.81 ± 1.93	2.31 ± 0.90	18.49 ± 2.15
6.0	5.05 ± 2.01	2.52 ± 1.20	19.38 ± 1.92
24.0	5.64 ± 2.20	2.95 ± 1.37	20.93 ± 2.45
48.0	5.87 ± 2.22	3.07 ± 1.43	21.75 ± 2.85
72.0	6.08 ± 2.23	-	-
144.0	6.47 ± 2.21	-	-

## Data Availability

Data are available upon request to the corresponding author.
